# Chronic IFN-γ Exposure Induces Divergent Adaptive Programs in Glioblastoma Subtypes

**DOI:** 10.3390/cancers18101552

**Published:** 2026-05-11

**Authors:** Elnaz Rahbarlayegh, Natsuko Nomura, Tiffany M. Juarez, Pranav R. Kesari, Santosh Kesari

**Affiliations:** 1 Pacific Neuroscience Institute, Santa Monica, CA 90404, USA; 2 The Lundquist Institute, Torrance, CA 90502, USA; 3 CureScience Institute, San Diego, CA 92121, USApranavkesari123@gmail.com (P.R.K.); 4 AsthraHealth, Inc., Santa Monica, CA 90405, USA

**Keywords:** interferon-γ, glioblastoma, STAT1, IRDS, PI3K–AKT, cytokine adaptation

## Abstract

Background: Immunotherapy has transformed cancer treatment by enhancing cytotoxic T-cell activity and interferon-γ (IFN-γ)-mediated tumor clearance. However, glioblastoma (GBM) remains largely refractory to these approaches, reflecting a profoundly immunosuppressive and myeloid-dominant tumor microenvironment. IFN-γ is central to antitumor immunity, yet chronic exposure can paradoxically promote adaptive resistance. How GBM cells respond to sustained IFN-γ signaling, and whether these responses differ across tumor states, remains poorly understood. Methods: To address this, we modeled chronic IFN-γ exposure in mesenchymal-like (U87) and proneural-like (U251) GBM cells over 28 days and performed integrated analyses of transcriptional, proteomic, and secretory responses. Results: While IFN-γ initially suppressed growth in both models, their long-term adaptations diverged. U87 cells developed a persistence-prone state characterized by progressive activation of PI3K–AKT signaling, whereas U251 cells exhibited sustained interferon signaling with persistent interferon-related DNA damage resistance signature (IRDS) expression and suppressed AKT activity. These transcriptional and signaling programs were incompletely reversible after cytokine withdrawal, indicating stable interferon conditioning. Analysis of TCGA glioblastoma datasets demonstrated that interferon-associated transcriptional programs are present across human tumors and are positively associated with PI3K–AKT pathway activity across molecular subtypes. Conclusions: Together, these findings reveal that chronic IFN-γ exposure drives distinct, lineage-dependent adaptive states in GBM, linking interferon signaling to divergent survival and immune-modulatory programs. While IFN-γ enhances immune activation, prolonged signaling may also promote tumor persistence. These results support therapeutic strategies that combine IFN-based approaches with interventions targeting adaptive survival pathways and immune reprogramming.

## Simple Summary

Interferon gamma (IFN-γ) is an immune signal that helps the body respond to cancer, but its long-term effects on tumor cells are not well understood. In this study, we examined how glioblastoma cells respond to prolonged exposure to this signal. Although both cell types initially showed similar immune responses, their long-term behavior differed. One cell type adapted and partially recovered, while the other developed a more persistent state with ongoing immune-related activity and altered growth. We also observed that key processes controlling cell growth and survival became less tightly coordinated over time. These findings suggest that continuous immune signaling can change how tumor cells behave in different ways depending on the context. A better understanding of these responses may help improve strategies that combine immunotherapy with other targeted treatments for glioblastoma.

## 1. Introduction

Immunotherapy has transformed the treatment landscape of several malignancies by harnessing the immune system to recognize and eliminate tumor cells. Checkpoint inhibitors, adoptive T-cell therapies, and cytokine-based approaches have shown durable clinical responses in melanoma, lung, and renal cancers by restoring cytotoxic T-cell activity and promoting interferon-γ (IFN-γ)-driven antitumor immunity [1,2,3,4]. Despite these advances, glioblastoma (GBM), remains largely refractory to immunotherapy.

GBM is the most aggressive primary brain malignancy, characterized by invasive growth, extensive heterogeneity, resistance to nearly all standard therapies and high mortality rate [5,6]. GBM tumor microenvironment is highly immunosuppressive, characterized by abundant myeloid and glial cells, low effector T-cell infiltration, and a network of tumor-derived cytokines and chemokines that polarize macrophages toward M2 phenotypes, recruit regulatory T cells, and inhibit natural killer (NK) cell cytotoxicity [7,8].

These factors generate a “cold” immune milieu that restrains antitumor immunity even when T-cell activity is pharmacologically enhanced. Immunotherapies primarily depend on activating T cells to produce IFN-γ, the key effector cytokine mediating tumor clearance, although in GBM this same pathway may paradoxically reinforce immune escape [9,10].

IFN-γ, secreted by activated T and NK cells, plays a central role in antitumor defense. Through the canonical JAK1/2–STAT1–IRF1 cascade, it induces antigen presentation via MHC-I upregulation, drives growth arrest and apoptosis, and enhances recognition of malignant cells [11,12,13]. However, sustained IFN-γ exposure can produce the opposite outcome, chronic activation of this pathway induces PD-L1 and IDO1 expression, promotes recruitment of suppressive myeloid cells, and fosters tolerance within the tumor niche [10,14,15]. These adaptive responses convert an initially inflammatory signal into a self-limiting, immunosuppressive loop.

Prolonged interferon signaling further establishes an interferon-conditioned state, characterized by persistent STAT1 phosphorylation and an Interferon-Related DNA-Damage Resistance Signature (IRDS) that confers resistance to radiation and temozolomide therapy [13,16]. Such transcriptional adaptation underlies the paradox of IFN-γ activity in tumors, while transient signaling promotes tumor elimination, chronic signaling stabilizes dormancy and therapy resistance [17,18]. In GBM patient samples, IFN-γ-associated gene expression frequently coincides with inhibitory checkpoints such as PD-L1 and TIM-3, defining an inflamed-yet-suppressed microenvironment that undermines the efficacy of T-cell-focused immunotherapies. Transcriptomic analyses have revealed substantial heterogeneity in interferon-associated gene expression across glioblastoma tumors, with subsets exhibiting elevated interferon signaling accompanied by distinct immune and inflammatory features [19,20]. These observations suggest that interferon activity in GBM reflects diverse tumor states rather than a uniform response. However, whether these interferon-associated programs correspond to specific tumor-intrinsic signaling adaptations, and how they relate to survival pathways within tumor cells, remains unclear. Understanding how GBM cells adapt to persistent IFN-γ signaling is therefore critical for interpreting the limited success of current immunotherapies. In this study, we modeled chronic IFN-γ exposure in two molecularly distinct GBM cell lines, mesenchymal-like U87 and proneural-like U251 to delineate the transcriptional, proteomic, and secretory adaptations that enable tumor persistence under sustained IFN-γ pressure.

## 2. Materials and Methods

### 2.1. Cell Culture

Human glioblastoma cell lines U87-MG (RRID:CVCL_0022) and U251-MG (RRID:CVCL_0021) were obtained from American Type Culture Collection (ATCC, Manassas, VA, USA) and maintained in Dulbecco’s modified Eagle’s medium (DMEM: Life Technologies, Carlsbad, CA, USA) supplemented with 10% fetal bovine serum (FBS: Life Technologies, Carlsbad, CA, USA) and 1% penicillin–streptomycin (Life Technologies, Carlsbad, CA, USA) in a humidified incubator at 37 °C with 5% CO_2_. Cells were thawed and experiments were initiated at passages 5–6. Cells were maintained in culture for up to four weeks prior to endpoint sampling, and no experiments were performed beyond passage 15. Cells were seeded at a density of 1 × 10^6^ cells per flask and passaged upon reaching 80–90% confluence, with the media replaced every two days. On D0, the cells and their media were collected from a T75 flask as the Untreated group, and the remaining groups were treated with 10 ng/mL IFN-γ (Life Technologies, Carlsbad, CA, USA) and media were replaced with fresh media containing 10 ng/mL IFN-γ every other day throughout the treatment period. The cells and conditioned media were collected at D3, D7, and D14 of IFN-γ (10 ng/mL) exposure; at D14, the cultures were split to either continue IFN-γ to D28 or washout (WO) into IFN-γ-free media, with additional collections at D21 and D28. Unless otherwise stated, biological replicates represent independent cell culture preparations initiated, maintained, and treated separately.

U87-MG and U251-MG are listed by ICLAC as commonly misidentified cell lines and were not independently authenticated by STR profiling after receipt. They were used here as established glioma models to examine IFNγ-driven signaling adaptation rather than to model patient-level glioblastoma heterogeneity.

### 2.2. Experimental Design

To evaluate the temporal effects of chronic IFN-γ exposure, a time-course experiment was designed in which samples were collected at distinct time points: 0, 3, 7, 14, 21, and 28 days. Each experimental group was analyzed in biological triplicates to ensure reproducibility. Specific time points were chosen based on preliminary studies showing differential RNA and protein responses. Notably, RNA changes were more evident at earlier time points (Day 3), whereas protein-level changes became significant at later time points (Day 7 onward). At the designated time points, the cell culture supernatant was collected and stored in aliquots at −80 °C. The cells were detached via trypsin-EDTA (Life Technologies, Carlsbad, CA, USA) for 3 min at 37 °C and washed three times with DMEM. The cells were either resuspended in freezing media (90% FBS + 10% DMSO) for storage at −80 °C or resuspended in DMEM for same-day use.

### 2.3. Microscopy and Morphometric Analysis

Morphological changes were evaluated by phase-contrast microscopy (Leica DMi8, Leica Microsystems, Wetzlar, Germany) at 300× magnification. Representative images were collected to document changes in cell shape, confluency, and growth patterns across conditions. These observations were qualitative and were not used for quantitative or statistical analysis.

Cell growth was quantified independently using total cell counts obtained during routine passaging and at defined experimental time points. Viable cells were counted using trypan blue exclusion and a hemocytometer.

### 2.4. RNA Isolation and Sequencing

Total RNA was extracted via the RNeasy Mini Kit (Qiagen, Hilden, Germany ) following the manufacturer’s protocol. RNA integrity was assessed via an Agilent Bioanalyzer 2100 (Agilent Technologies, Santa Clara, CA, USA), with an RNA integrity number (RIN) threshold of >8. Libraries were prepared via the TruSeq RNA Library Prep Kit (Illumina, CA, USA) and sequenced on an Illumina HiSeq 2500 platform (Illumina, San Diego, CA, USA) to generate 150 bp paired-end reads. The sequence depth was maintained at a minimum of 50 million reads per sample to ensure adequate coverage. Quality control of the raw reads was performed via FastQC (v0.11.9), and adapter trimming was conducted via Trim Galore (v0.6.6). Read alignment and quantification were performed via STAR (v2.7.10a) and FeatureCounts (v2.0.3) against the GRCh38 reference genome. Lowly expressed genes were filtered and library-size normalization was performed with edgeR (version 4.3.1), and differential gene expression analysis was then carried out using the voom–limma pipeline; genes with a false discovery rate (FDR) < 0.05 and greater than a two-fold change in expression (log_2_ fold change > 1) were considered significant [21]. All downstream analyses and visualizations (heatmaps, volcano plots, Hallmark pathway scoring, interferon-module scoring, and gene set enrichment analyses) were performed in R (version 4.3.1).

### 2.5. Protein Extraction and Automated Western Blotting (WES)

Proteins were extracted via RIPA buffer (Thermo Scientific, Thermo Fisher Scientific, Waltham, MA, USA) containing protease and phosphatase inhibitors. Protein concentrations were quantified via the Enhanced BCA Protein Assay Kit (Thermo Scientific, Thermo Fisher Scientific, Waltham, MA, USA) according to the manufacturer’s protocol. Protein samples were standardized to a concentration of 0.5 μg/μL for analysis. Automated western blotting was conducted via the WES system (ProteinSimple, San Jose, CA, USA). The antibodies used for detection included phospho-Stat1 (Tyr701) (58D6) (Cell Signaling Technology, Danvers, MA, USA; rabbit mAb #9167S, dilution 1/50), phospho-Stat3 (Tyr705) (D3A7) (Cell Signaling Technology, MA, USA; rabbit mAb #9145S, dilution 1/10), phospho-Akt (ser473) (D9E) (Cell Signaling Technology, MA, USA; rabbit mAb #4060S), rabbit mAb #4370S, dilution 1/50), and phospho-S6 ribosomal protein (ser235/236) (D57.2.2E) (Cell Signaling Technology, Danvers, MA, USA; rabbit mAb #4858S). Signal intensities were quantified via Compass software(v6.0.0, ProteinSimple, San Jose, CA, USA).

### 2.6. Single-Cell Proteomic Analysis

Analysis was limited to comparing Untreated group with day 14; which represented the most informative time point based on transcriptomic and bulk signaling alterations. Single-cell proteomic analyses were performed using the IsoCode Tumor Signaling platform (IsoPlexis, Branford, CT, USA; now PhenomeX) to assess heterogeneity in intracellular signaling.

Cells were detached, washed in PBS, and stained with CellTrace FarRed (1:10,000 dilution; Life Technologies, Carlsbad, CA, USA) for 15 min at 37 °C in the dark. Following staining, cells were quenched with complete medium, washed, and resuspended. Cell suspensions were filtered through a 70 μm filter to remove aggregates prior to chip loading. Cells were then resuspended at 1 × 10^6^ cells/mL, and 35 μL of suspension (~35,000 cells) was loaded per condition onto Human IsoCode Tumor Signaling Chips and processed using the IsoLight instrument (IsoPlexis, Branford, CT, USA).

Single-cell measurements were obtained from captured and quality-filtered cells as determined by the IsoSpeak analysis pipeline (v2.8.0.0; IsoPlexis, Branford, CT, USA). The number of analyzable cells per condition was as follows: U87 untreated (*n* = 597), U87 IFN-γ (14 days) (*n* = 432), U251 untreated (*n* = 589), and U251 IFN-γ (14 days) (*n* = 744). Proteomic profiles were normalized across biological replicates, and statistical significance was assessed using the Mann–Whitney U test.

Functional heterogeneity was quantified using the functional heterogeneity index (FHI), a composite metric generated by IsoSpeak (v2.8.0.0) that summarizes the diversity and co-occurrence of functional protein modules across individual cells within a population. Higher FHI values indicate increased functional heterogeneity and multiplexed signaling states rather than uniform pathway activation.

Single-cell correlation networks were generated in IsoSpeak by computing pairwise correlations between phosphorylated signaling proteins across individual cells within each condition. These correlations reflect coordinated phosphorylation patterns across heterogeneous cell populations and do not imply direct molecular interactions or causal relationships. Only statistically significant correlations, as determined by the IsoSpeak analysis pipeline, were visualized.

### 2.7. Secretome Profiling

The cryopreserved supernatants were thawed at room temperature for 30–60 min and mixed thoroughly by pipetting. An aliquot of 5.5 μL from each sample was loaded into the macro chambers of a CodePlex Human Adaptive Immune Panel chip, prepatterned with a 23-plex antibody array. A 2% BSA/PBS mixture was used as background control. The chip was subsequently processed in the IsoLight automation system, and cytokine levels were quantified via fluorescence-based ELISA. The data were analyzed via IsoSpeak software (v2.8.0.0).

### 2.8. Statistical Analysis

All experiments were performed using independent biological triplicates (*n* = 3). Statistical analyses were conducted in R (version 4.3.1; R Core Team, Vienna, Austria) using packages for differential expression (edgeR, limma), pathway enrichment (msigdbr, fgsea), and data visualization (ggplot2, ComplexHeatmap). Data normality was assessed using the Shapiro–Wilk test prior to parametric analyses, and no significant deviations from normality were detected. No formal statistical test for outliers was conducted, and no data points were excluded from the analyses.

Pairwise comparisons were performed using two-tailed Student’s *t* tests, while multiple group comparisons were analyzed by one-way ANOVA followed by Tukey’s post hoc test. Statistical significance was defined as *p* < 0.05, with adjusted *p* values calculated where appropriate to account for multiple comparisons. No a priori statistical power or sample size calculation was performed; sample sizes were determined based on prior studies using similar experimental designs and feasibility considerations associated with extended time-course and multi-omics analyses, as indicated in the figure legends.

### 2.9. TCGA Data Acquisition and Computational Analysis

Gene expression data for glioblastoma (GBM) tumors (*n* = 172) were obtained from The Cancer Genome Atlas (TCGA) cohort HiSeqV2 platform (Illumina, San Diego, CA, USA). The dataset consisted of preprocessed normalized gene expression values. Expression values were log2-transformed (log2(x + 1)) prior to analysis.

Gene expression values were standardized across tumors for each gene (row Z-score normalization).

To evaluate interferon-associated transcriptional programs, gene sets were defined using both curated Molecular Signatures Database (MSigDB) pathways (including Hallmark and Reactome gene sets) and custom gene modules based on literature-supported signatures, including interferon response (IRDS), antigen presentation, immune checkpoint, and immune cell-associated programs.

Pathway scores were calculated as the mean gene-wise Z-score of genes within each pathway for each tumor. Gene sets with fewer than three genes represented in the dataset were excluded from scoring.

Associations between IFN-γ signaling, PI3K–AKT pathway activity, and immune-related gene modules were evaluated using Spearman correlation analysis. Where indicated, tumors were stratified by transcriptional subtypes based on available TCGA annotations. Statistical significance was defined as *p* < 0.05.

All analyses and visualizations were performed in R (version 4.3.1) using packages including ggplot2 and ComplexHeatmap.

Full statistical results for all quantitative assays are provided in Supplementary Tables S1–S4.

## 3. Results

### 3.1. IFN-γ Induces Distinct, Time-Dependent Growth Responses in GBM Cells

**Figure 1:**
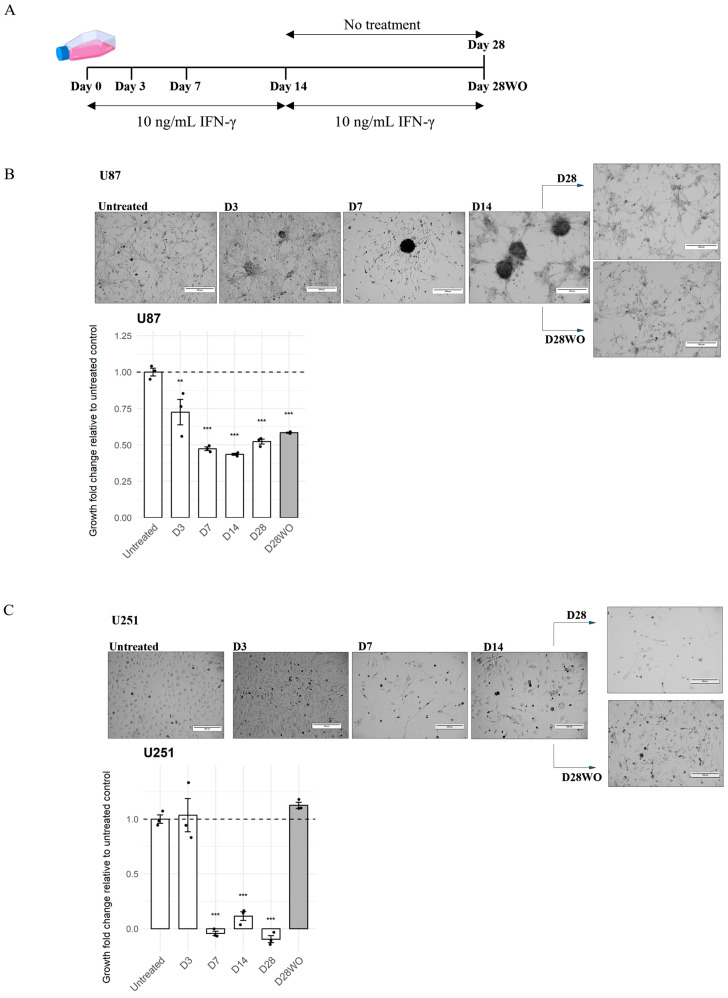
Morphological and growth responses of human glioblastoma cell lines to chronic IFN-γ exposure. (**A**) Schematic illustration of the treatment timeline. U87 and U251 cells were exposed to 10 ng/mL IFN-γ for 14 days (D0–D14), followed either by continued IFN-γ exposure until Day 28 or without treatment (D28 WO). (**B**) Representative phase-contrast images of U87 cells at the indicated time points. IFN-γ exposure led to progressive cluster formation and increased intercellular spacing, which partially reversed following cytokine withdrawal. The lower panel shows the relative growth rate normalized to untreated controls at D0 (mean ± SEM, *n* = 3 independent biological replicates). Statistical significance compared to untreated cells is indicated (** *p* < 0.01, *** *p* < 0.001). (**C**) Representative phase-contrast images of U251 cells at the same time points. IFN-γ treatment caused a marked decrease in cell density and elongation of cellular processes, followed by partial recovery after washout. The lower panel shows relative growth rate normalized to untreated controls (mean ± SEM, *n* = 3 independent biological replicates). Statistical significance compared to untreated cells is indicated (** *p* < 0.01, *** *p* < 0.001). Images are shown to illustrate morphology and relative confluence; quantitative comparisons of proliferation are based on cell counts. Scale bars, 300 µm.

To evaluate the direct impact of IFN-γ on glioblastoma cell growth, U87 and U251 cells were exposed to IFN-γ (10 ng/mL) for up to 28 days, with or without cytokine washout (Figure 1A–C). Proliferation was quantified using longitudinal cell counts across time points, from which relative growth and doubling time were derived.

Notably, visual assessments of confluence did not consistently reflect quantified proliferation rates, particularly in U251 cells, whose thin and elongated morphology limited visual discrimination of cell density across treatment conditions.

IFN-γ treatment was associated with reduced relative growth in both cell lines compared with untreated controls (*p* < 0.01; Supplementary Table S1).

While doubling time increased in both models at early time points, this metric could not be consistently applied under conditions where cell numbers remained static or decreased, particularly in U251 cells during prolonged IFN-γ exposure. Accordingly, relative growth provided a more robust measure of proliferative dynamics across the full-time course.

The temporal pattern of growth response differed between models. U87 cells exhibited a sustained reduction in growth beginning at D3 (*p* < 0.01), with only partial recovery following cytokine washout. In contrast, U251 cells showed minimal change at D3 but progressive growth suppression during chronic IFN-γ exposure, followed by marked rebound after washout, restoring or exceeding baseline growth levels (*p* < 0.001).

These findings indicate that IFN-γ imposes an early constraint on GBM cell growth while driving cell line-specific and differentially reversible proliferative responses.

### 3.2. IFN-γ Elevates STAT1 in Both GBM Lines but Elicits Opposite AKT Trajectories

**Figure 2:**
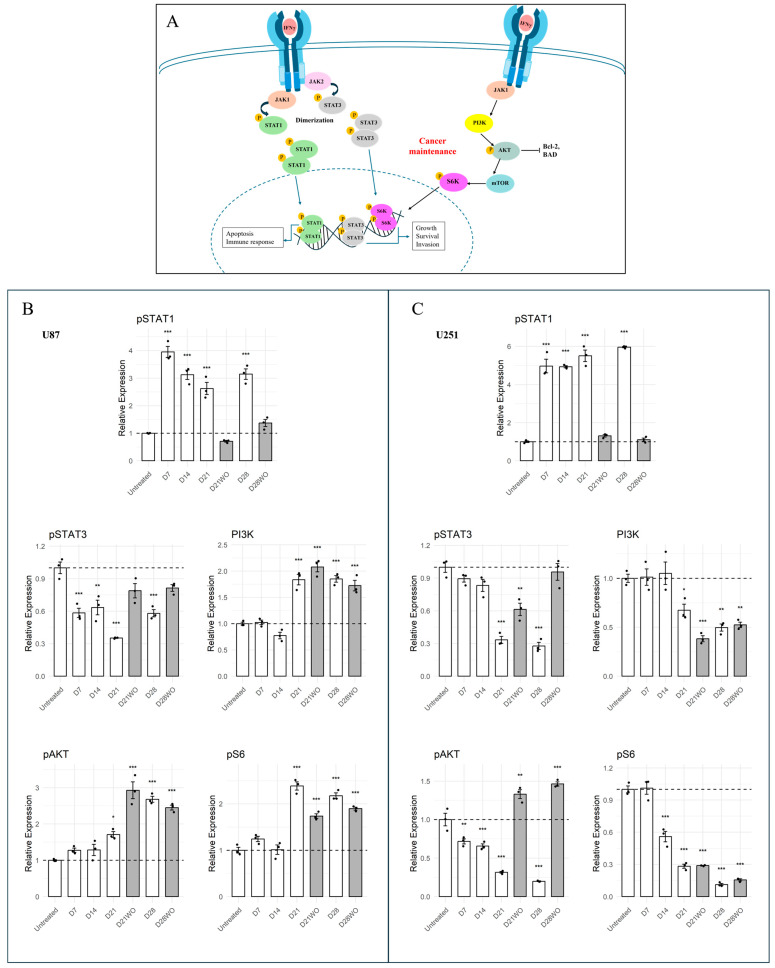
IFN-γ signaling differentially modulates JAK–STAT and AKT–mTOR pathways in U87 and U251 glioblastoma cells. (**A**) Schematic representation of IFN-γ receptor-mediated signaling illustrating JAK1/2 activation, STAT1/3 phosphorylation, and downstream interaction with the PI3K–AKT–mTOR–S6 axis regulating cellular growth and survival. (**B**) Quantification of phosphorylated STAT1, STAT3, AKT, and S6 in U87 cells at the indicated time points. Protein levels were by automated capillary Western blotting (WES) using equal protein input and expressed relative to untreated controls at D0 (mean ± SEM, *n* = 3 independent biological replicates). IFN-γ exposure led to robust induction of pSTAT1, whereas pAKT and pS6 showed an initial reduction during early exposure followed by increased signal at later time points, indicating dynamic regulation of growth-associated signaling under prolonged cytokine stimulation. (**C**) Corresponding analysis in U251 cells under the same treatment paradigm (D3–D28 ± washout). Chronic IFN-γ exposure resulted in sustained elevation of pSTAT1, while pAKT, pS6, and STAT3-associated readouts were reduced during prolonged exposure, with partial recovery of AKT- and STAT3-associated signaling following cytokine withdrawal. Data are shown as the mean ± SEM; *n* = 3 independent biological replicates. Statistical significance versus untreated controls is indicated. Phosphorylated STAT1, STAT3, and AKT signals were normalized to their corresponding total protein levels. For S6, only the phosphorylated form was measured; thus, changes reflect phosphorylation dynamics rather than total protein abundance. PI3K is presented as total protein levels. Statistical significance compared to untreated controls is indicated as follows: * *p* < 0.05, ** *p* < 0.01, *** *p* < 0.001. Representative pseudoblot images generated from automated capillary-based Western blot analysis are provided in Supplementary Materials.

To characterize intracellular signaling responses to IFN-γ in the two cell lines, we examined the phosphorylation levels and expression of key proteins involved in growth and survival pathways. Figure 2A summarizes the IFN-γ receptor–JAK1/2–STAT signaling pathway and its crosstalk with the PI3K–AKT–mTOR signaling axis. STAT1 phosphorylation increased in both U87 and U251 cells during IFN-γ exposure, with distinct temporal patterns. In U87 cells, phosphorylated STAT1 was significantly elevated by day 7 relative to untreated cells (*p* < 0.001) and remained above baseline throughout the treatment course, with a modest decline from the day 7 peak. In U251 cells, phosphorylated STAT1 increased progressively over time (*p* < 0.001), reaching maximal levels at later time points. In both cell lines, pSTAT1 levels returned toward baseline following IFN-γ washout.

PI3K–AKT–mTOR pathway readouts diverged between the two cell lines. In U87 cells, PI3K protein levels showed a modest decrease at early time points followed by increased expression during prolonged IFN-γ exposure, paralleling the increase in phosphorylated AKT and phosphorylated S6 observed at later time points (*p* < 0.001), which persisted following cytokine washout. In contrast, U251 cells exhibited relatively stable PI3K levels during early exposure with a decrease at later time points, accompanied by progressive suppression of AKT pathway phosphoproteins during IFN-γ exposure, becoming most pronounced after 14 days of treatment. Following washout, pAKT levels showed partial recovery, whereas pS6 demonstrated only modest recovery, indicating a distinct signaling trajectory under identical treatment conditions (Figure 2B,C).

Changes in STAT1, STAT3, and AKT phosphorylation were quantified relative to their corresponding total protein levels, whereas changes in PI3K and pS6 are presented as relative expression or phosphorylation without normalization to corresponding total protein levels. Importantly, recovery of proliferative capacity after IFN-γ washout did not consistently coincide with restoration of these signaling readouts, suggesting partial uncoupling between proliferation recovery and PI3K–AKT–mTOR pathway activity as assessed by these markers.

### 3.3. Temporal Gene Expression and Pathway Enrichment in GBM Cells

To characterize the temporal interferon transcriptional response, we analyzed a focused set of IFN-γ-responsive genes representing core signaling (IFNGR1/2, JAK1/2, STAT1, IRF1), antigen presentation (CIITA/NLRC5, HLA class I/II, TAP1/2, TAPBP, B2M), immunoproteasome components (PSMB8/9/10, PSME1/2), chemokines (CXCL9/10/11, CCL2/CCL5), and negative feedback regulators (SOCS1/3, USP18, PTPN1/2). Gene expression is presented as row z-scored group means across all conditions.

#### 3.3.1. U87 Cells Display a Two-Phase on–off–on Response with Delayed Upregulation of Chemokines

**Figure 3:**
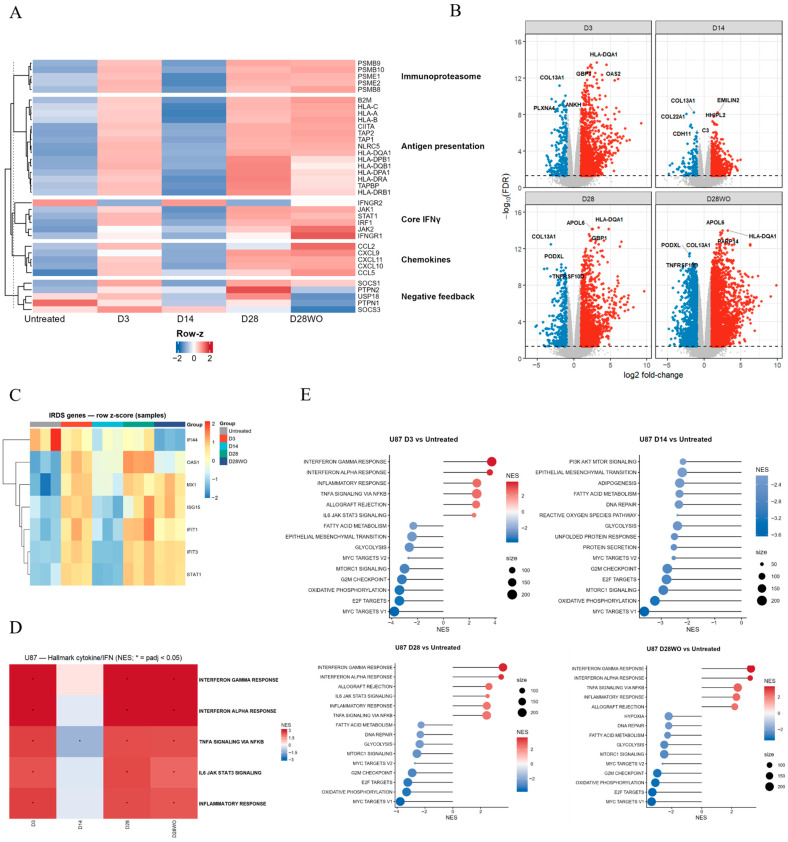
Transcriptomic responses of U87 glioblastoma cells to chronic IFN-γ exposure. (**A**) Heatmap of representative IFN-γ-responsive genes categorized into functional groups including immunoproteasome, antigen presentation, core IFN-γ signaling, chemokines, and negative feedback regulators across time points (Untreated, D3, D14, D28, D28WO, n = 3 independent biological replicates for each). Gene expression values are shown as row Z scores of normalized counts. (**B**) Volcano plots showing differential gene expression at each indicated time point relative to Untreated cells. Selected significantly upregulated and downregulated genes are labeled. (**C**) Heatmap of interferon-related DNA damage resistance signature (IRDS) genes in U87 cells across time points, shown as row Z scores of expression values. (**D**) Heatmap summarizing the Hallmark cytokine/IFN-related pathways (GSEA; normalized enrichment score, NES) significantly enriched (*p*adj < 0.05) during the course of IFN-γ treatment and withdrawal. (**E**) Gene set enrichment analysis (GSEA) of top Hallmark pathways comparing each indicated time point (D3, D14, D28, D28WO) versus Untreated. Plots display normalized enrichment scores (NES) and gene set sizes, highlighting dynamic activation of IFN-γ, TNF-α, and JAK–STAT-related signatures over time. For heatmaps and enrichment analyses, red indicates relatively high expression or enrichment, whereas blue indicates relatively low expression or enrichment. In volcano plots, red and blue points represent significantly upregulated and downregulated genes, respectively, while gray points indicate non-significant genes.

We first examined transcriptional responses in U87 cells following chronic IFN-γ exposure. At day 3, core IFNγ signaling and antigen presentation genes were strongly induced (Figure 3A). This early activation was followed by a relative attenuation at day 14, interrupting the initial response and preceding reactivation at later time points (i.e., day 28, with and without IFN-γ), consistent with a non-linear, on–off–on trajectory.

Chemokine expression displayed delayed kinetics. CXCL9/10/11 and CCL2/5 showed only modest induction at early time points but reached maximal expression at day 28 (with and without IFN-γ) (Figure 3A). Immunoproteasome components followed a similar activation–attenuation–reactivation pattern. In contrast, negative feedback regulators accumulated progressively during IFN-γ exposure and remained partially elevated after washout.

Pathway-level analysis recapitulated these gene-level trends. Interferon response pathways (INTERFERON-γ/α RESPONSE), along with TNFα–NF-κB and IL6–JAK–STAT signaling, peaked early, showed relative attenuation at day 14, and re-emerged at later time points (Figure 3B,C). In contrast, growth and metabolic programs (MYC/E2F, G2M, mTORC1, OxPhos) remained consistently suppressed throughout the time course (Figure 3D,E).

#### 3.3.2. U251 Cells Display a Sustained Interferon Program with Strong Late Chemokines and Feedback

**Figure 4:**
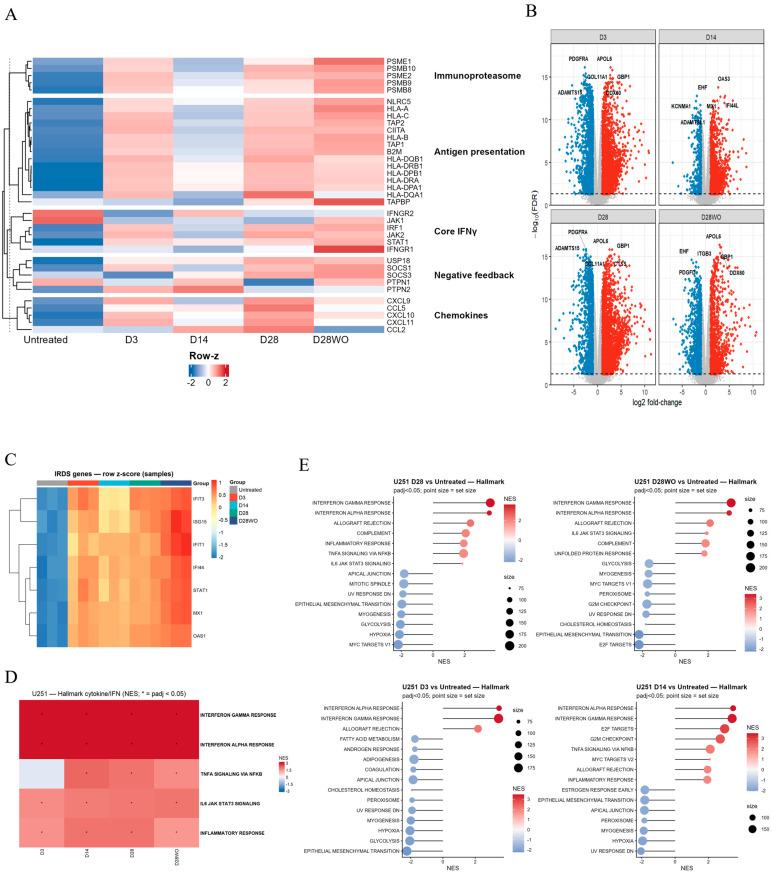
Transcriptomic responses of U251 glioblastoma cells to chronic IFN-γ exposure. (**A**) Heatmap of representative IFN-γ-responsive genes grouped into immunoproteasome, antigen presentation, core IFN-γ signaling, negative feedback regulators, and chemokines across time points (Untreated, D3, D14, D28, D28WO, *n* = 3 independent biological replicates for each). Values are row Z scores of normalized expressions. (**B**) Volcano plots of differential gene expression at each indicated time point versus Untreated. X-axis: log2-fold change; Y-axis: –log10(FDR). Selected significantly up- and downregulated genes are labeled. (**C**) Heatmap of IRDS (interferon-related DNA damage resistance signature) genes across time points, shown as row Z scores. (**D**) Heatmap summarizing Hallmark cytokine/IFN-related pathways that are significantly enriched (NES, *p*adj < 0.05) over the course of treatment and withdrawal. (**E**) GSEA/Hallmark results for D3, D14, D28, and D28WO versus Untreated. Bubble plots display normalized enrichment scores (NESs) and gene set sizes for the top enriched pathways at each comparison. For heatmaps and enrichment analyses, red indicates relatively high expression or enrichment, whereas blue indicates relatively low expression or enrichment. In volcano plots, red and blue points represent significantly upregulated and downregulated genes, respectively, while gray points indicate non-significant genes.

We next analyzed transcriptional responses in U251 cells under the same treatment conditions. In contrast to U87, U251 cells exhibited sustained activation of core IFNγ signaling and antigen presentation programs across all time points, with only partial attenuation following IFN-γ washout (Figure 4A).

Immunoproteasome gene expression increased progressively and reached maximal levels at day 28, with or without IFN-γ washout. Chemokine expression was detectable as early as day 3 and intensified further at later time points, resulting in strong expression at day 28 and after washout (Figure 4A). Negative feedback regulators accumulated steadily and remained prominent at late time points.

Consistent with these observations, IRDS-associated genes remained elevated throughout the time course (Figure 4C), and pathway analysis confirmed sustained activation of INTERFERON-γ/α RESPONSE signatures, together with co-activation of TNFα–NF-κB and IL6–JAK–STAT3 pathways during IFN-γ exposure (Figure 4D).

Cell cycle-associated gene sets (E2F, G2M, MYC targets) exhibited a modest transient rebound at day 14 before declining again at later time points. Following washout, interferon signaling remained detectable, and most metabolic and plasticity-associated pathways remained suppressed (Figure 4E).

To assess whether interferon-driven programs extend to immune checkpoint regulation, we examined CD274 (PD-L1) and IDO1 expression. Both genes were induced in response to IFN-γ in both models but exhibited distinct temporal patterns. U87 cells showed progressive CD274 upregulation with delayed induction of IDO1. In contrast, U251 cells exhibited higher baseline expression and markedly stronger, sustained induction of both genes (Figure S3), consistent with the persistence of interferon signaling in this model.

Together, these results demonstrate that both cell lines activate robust interferon, antigen presentation, and immunoproteasome programs, but with distinct temporal organization. U87 cells exhibit a non-linear temporal response with delayed chemokine dominance, whereas U251 cells maintain a sustained interferon/IRDS state characterized by strong feedback regulation and late chemokine amplification. In both models, signaling programs are not fully restored after washout, indicating an incompletely reversible IFNγ-conditioned state. Gene-level differential expression statistics are provided in Supplementary Tables S3 and S4.

### 3.4. IFN-γ Expands Highly Coactivated Single-Cell States and Reshapes the Wiring of Signaling Networks

**Figure 5:**
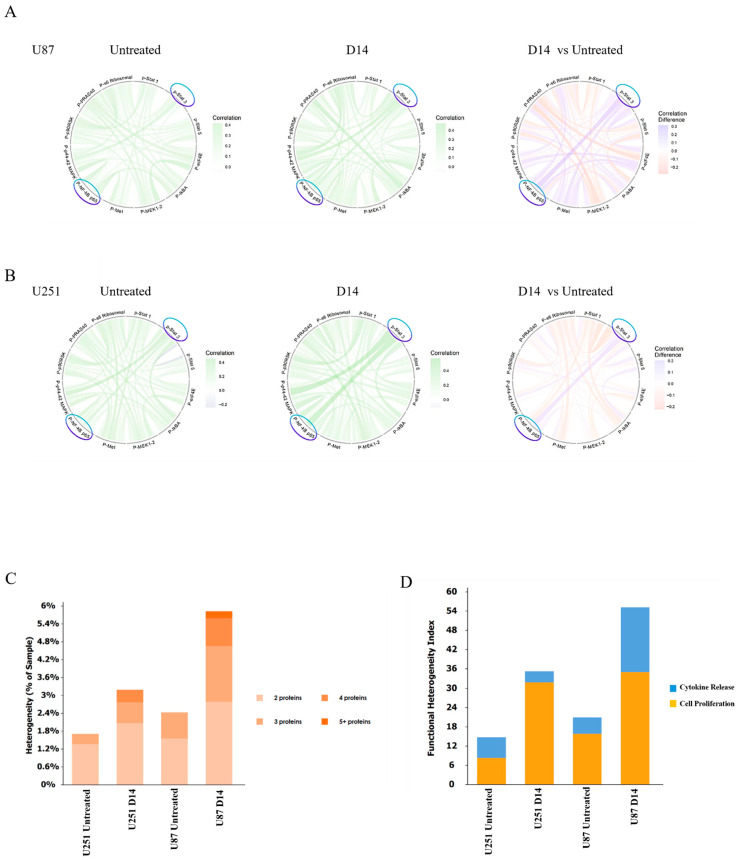
Protein network correlation and functional heterogeneity under chronic IFN-γ exposure. (**A**) Circos correlation maps showing statistically significant pairwise correlations among phosphorylated proteins involved in IFN-γ-related signaling (p-STAT1, p-STAT3, p-Akt, p-mTOR, p-S6, and p-Erk1/2) measured at the single-cell level in Untreated and Day 14 (D14) U87 cells. Pairwise correlations were computed across individual cells using the IsoSpeak analysis pipeline, and only statistically significant correlations were visualized. Connections represent coordinated phosphorylation behaviors across individual cells rather than direct molecular interactions or causal signaling relationships. Green ribbons indicate positive correlations, while purple ribbons mark negative correlations. The D14 vs Untreated panel displays correlation differences, with pink tones indicating stronger and blue tones weaker correlations after IFN-γ exposure. Analysis was performed on quality-filtered single cells (n = 597 for untreated and n = 432 for D14). (**B**) U251 glioblastoma cells. Equivalent Circos correlation plots showing single-cell phosphoprotein covariation patterns in Untreated and D14 samples and the corresponding D14 vs Untreated differential map. Both cell lines show remodeling of signaling-protein relationships after IFN-γ treatment, reflecting altered pathway coordination. Analysis was performed on quality-filtered single cells (*n* = 589 for untreated and *n* = 744 for D14). (**C**) Stacked bar plots summarizing the distribution of protein coregulation heterogeneity, expressed as the percentage of samples exhibiting coordinated variation among 2, 3, 4, or ≥5 proteins. Chronic IFN-γ exposure increases multiprotein coregulation, most notably in U87 cells. (**D**) Bar plots showing the functional heterogeneity index (FHI), a composite metric generated by the IsoSpeak software, partitioned by cytokine-release (blue) and cell-proliferation (orange) categories. Both cell lines demonstrate higher functional heterogeneity following IFN-γ exposure, with a stronger contribution from proliferation-associated proteins in U87 cells. FHI values were derived from single-cell measurements across the analyzed cell populations.

Phosphoprotein levels were quantified across thousands of individual cells per condition, and pairwise correlations between signaling markers were calculated to capture coordinated signaling behaviors within heterogeneous cell populations. After two weeks of IFN-γ exposure (day 14), both the magnitude of individual signaling markers and their coordinated behavior within single cells were altered. In both U87 and U251 cells, the Day 14 chord maps appeared denser and more interconnected than at baseline, and the Day 14 versus untreated difference plots revealed more increased positive correlations than losses (Figure 5A,B). In these chord maps, connections represent statistically significant positive or negative correlations between phosphoproteins across single cells and do not imply direct molecular interactions or causal signaling relationships. The STAT3–NF-κB relationship differed across conditions: it was present at baseline in both cell lines and became most pronounced in U251 cells at Day 14. The broader phosphoprotein pattern differed by model. U87 showed a general tightening of the network, with many correlations becoming stronger, whereas U251 displayed more selective strengthening, with a smaller set of connections, including STAT3–NF-κB, becoming more prominent.

Consistent with ensemble-averaged phosphoprotein measurements obtained by automated capillary immunoblotting (WES), single-cell analysis revealed coordinated shifts in phosphorylation patterns and correlation structure rather than mutually cancelling subpopulations, indicating that dominant signaling programs are shared across substantial fractions of cells.

The composition of single cells producing phosphorylated proteins also shifted. After IFN-γ exposure, more cells produced several phosphoproteins simultaneously, and this multiplex-positive fraction increased in both lines, most notably in U87 at Day 14 (Figure 5C). The functional heterogeneity index (FHI) increased from the untreated state to Day 14 in each model (Figure 5D), consistent with the time-dependent separation of transcriptomic profiles. While early time points clustered tightly, replicates from prolonged IFN-γ exposure (Day 28 and Day 28 washout) displayed greater dispersion, indicating increased transcriptional heterogeneity within the U87 population (Figure S1D). In contrast, U251 replicates clustered together at all time points (Figure S2D). In U251 cells, the FHI increase was driven primarily by the cell proliferation module, whereas U87 cells exhibited a broader increase that included a substantial cytokine release component.

### 3.5. Cytokine Secretion Shifts with IFN-γ and Differs by Cell Line

**Figure 6:**
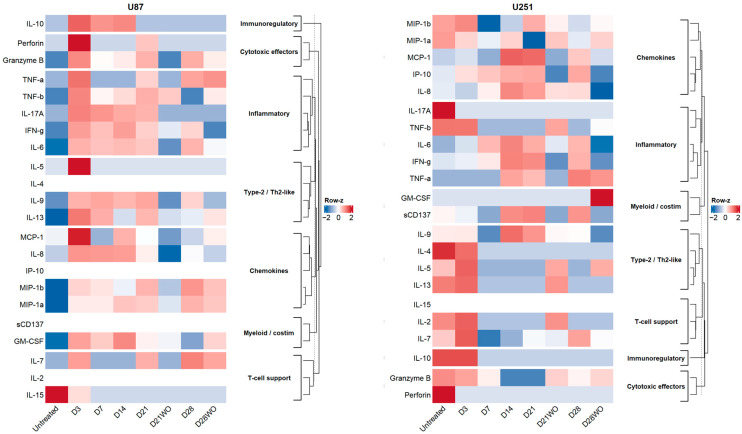
Cytokine secretion dynamics of U87 and U251 glioblastoma cells during chronic IFN-γ exposure and withdrawal. Heatmap showing cytokine concentrations measured in the culture supernatants of U87 and U251 cells across the experimental time points, following 10 ng/mL IFN-γ treatment. Both cell lines display mosaic, time-dependent secretion patterns rather than uniform increases or decreases. In U87 cells, transient elevations in several cytokines (e.g., IL-17A, IL9, IL-10) appear during mid-treatment, followed by partial normalization after washout. U251 cells show distinct modulation of TNF-family cytokines, chemokines (e.g., MCP-1/CCL2), and interleukins, with alternating activation and suppression phases. Overall, the heatmap highlights dynamic and heterogeneous cytokine responses to prolonged IFN-γ stimulation, reflecting differential adaptive behavior between the two glioblastoma lines.

To characterize the effect of IFN-γ on cytokine production, the CodePlex Human Adaptive Immune Panel was used to quantify the frequencies of cytokines secreted from U87 and U251 cells at designated time points (Figure 6). In U87 cells, IFN-γ exposure increased several inflammatory/chemotactic readouts, including TNF-α, MIP-1β (CCL4), IL-13, IL-10, and Granzyme B, whereas IL-15 tended to decrease, an overall pattern that persisted into later time points with partial relaxation after washout. In U251 cells, the trajectory was distinct: TNF-α and MCP-1 (CCL2) levels increased with treatment, whereas multiple interleukins linked to T-cell/Th2 signaling (IL-2, IL-4, IL-5, IL-10, and IL-13) generally tended to decrease; these factors spiked early (day 3) and then diminished. Notably, GM-CSF showed a late increase in U251 cells, which was prominent on day 28, with IFN-γ washout. Together, the secretome data indicates that IFN-γ pushes U87 toward a broadly pro-inflammatory, polycytokine output, whereas U251 cells exhibit a more selective program with sustained TNF-α/CCL2 upregulation and attenuation of several lymphokines. An integrated summary of transcriptional, signaling, and functional responses across both models, including temporal context, is provided in Supplementary Table S5.

### 3.6. Clinical Relevance of Interferon-Associated Adaptive Programs in Human Glioblastoma

**Figure 7:**
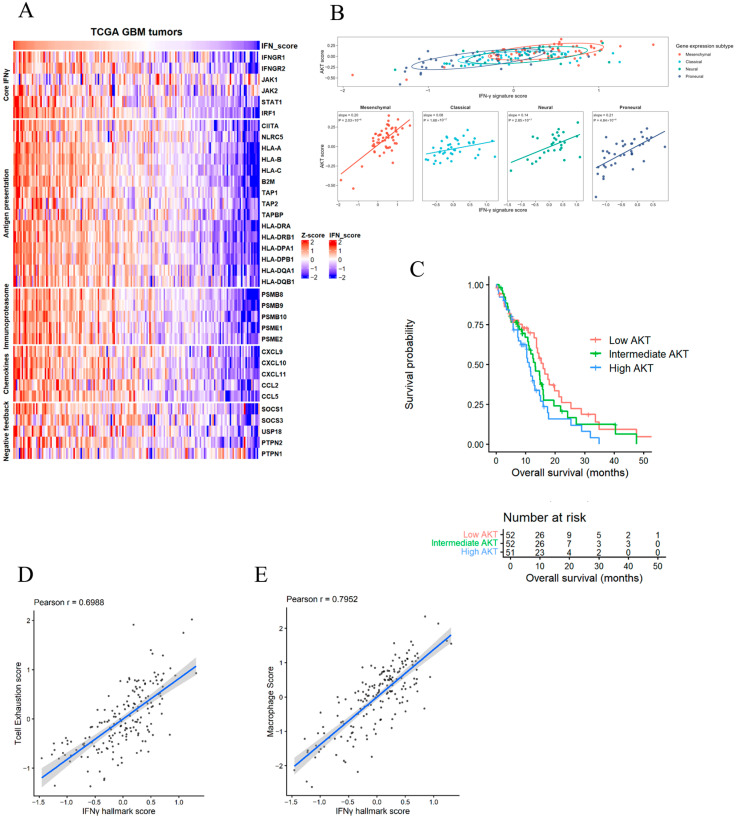
Interferon-associated transcriptional programs in human glioblastoma are linked to survival signaling, immune context, and clinical outcome. (**A**) Heatmap of interferon-associated gene expression across TCGA glioblastoma tumors. A curated IFN-γ response panel comprising core signaling (IFNGR1/2, JAK1/2, STAT1, IRF1), antigen presentation, immunoproteasome, chemokine, and negative feedback genes is shown. Gene expression values were standardized across tumors (row Z-score), revealing coordinated variation and substantial heterogeneity in interferon-associated transcriptional programs. (**B**) Association between interferon signaling and PI3K–AKT pathway activity. Top: global relationship between IFN-γ signature score (derived from the curated gene panel) and AKT pathway score across all tumors. Bottom: subtype-stratified analyses demonstrate consistent positive associations across mesenchymal, classical, neural, and proneural glioblastoma subtypes. (**C**) Kaplan–Meier analysis of overall survival stratified by AKT pathway activity. Tumors were grouped into tertiles based on AKT score (low, intermediate, high; *n* = 52, 52, 51, respectively). Higher AKT pathway activity was associated with reduced overall survival, although this did not reach statistical significance (log-rank *p* = 0.093). (**D**) Correlation between IFN-γ signaling and T-cell exhaustion-associated gene expression. Increased IFN-γ activity is associated with enrichment of T-cell exhaustion signatures (Pearson r = 0.70). (**E**) Correlation between IFN-γ signaling and macrophage-associated gene expression. Elevated IFN-γ signaling is associated with increased macrophage-related signatures (Pearson r = 0.80).

To assess whether the interferon-associated programs identified in our experimental models are reflected in human tumors, we analyzed gene expression data from TCGA glioblastoma samples. Interferon-related genes exhibited substantial heterogeneity across tumors, with coordinated variation in core IFN-γ signaling, antigen presentation, immunoproteasome, chemokine, and negative feedback modules, indicating the presence of distinct interferon-associated transcriptional states in human GBM (Figure 7A).

We next examined the relationship between interferon signaling and tumor-intrinsic survival pathways. Across the TCGA cohort, IFN-γ pathway activity was positively associated with PI3K–AKT signaling, and this relationship was preserved across transcriptional subtypes, supporting a link between interferon-associated programs and survival signaling across diverse GBM contexts (Figure 7B). Additionally, Kaplan–Meier analysis showed a trend toward reduced overall survival in tumors with elevated PI3K–AKT pathway activity compared with AKT-low tumors, although this did not reach statistical significance (log-rank *p* = 0.093; Figure 7C). To define the immune context associated with interferon signaling, we evaluated correlations between IFN-γ activity and immune-related gene modules. Higher IFN-γ signaling was associated with increased macrophage abundance and with enrichment of T-cell exhaustion-associated signatures, consistent with an inflamed yet functionally suppressed immune microenvironment (Figure 7D,E). Together, these findings indicate that interferon-associated transcriptional programs identified in vitro are recapitulated in human glioblastoma and are linked to survival signaling and immune context.

## 4. Discussion

The limited and often transient responses of glioblastoma to immunotherapy suggest that immune effector pathways, though activated, are functionally rewired within the tumor context. This study argues that the clinical fate of IFN-γ-based or IFN-priming strategies in GBM depends less on the presence of interferon signaling per se than on how lineage state and network context channel chronic IFN-γ.

IFN-γ is a central inflammatory cue in GBM that canonically activates JAK/STAT1, antigen presentation, and interferon-stimulated genes (ISGs), supporting antitumor immunity [22,23]. Genetic loss of IFN-γ signaling in host or tumor cells accelerates tumor growth and blunts rejection [24,25], yet prolonged activation of the same pathway can induce PD-L1 and IDO1 via STAT1–IRF1, creating an inflamed-yet-suppressed milieu observed in glioma models [10,15]. Consistent with these findings, our temporal analysis indicates that chronic IFN-γ exposure does not sustain maximal pathway activation but instead follows a non-linear trajectory, in which early transcriptional induction is followed by a transient attenuation phase before stabilization of adaptive programs. Similar intermediate reprogramming states have been described under prolonged interferon exposure in cancer systems, where sustained signaling drives transcriptional remodeling and resistance-associated phenotypes [26,27]. Both U87 and U251 cells in our study initiated a canonical interferon backbone marked by STAT1 activation and upregulation of antigen-processing and immunoproteasome components, confirming preserved interferon competence. However, their downstream adaptations diverged sharply, revealing lineage-dependent models of interferon adaptation. This divergence also extended to checkpoint-associated transcriptional outputs, as chronic IFN-γ induced CD274 and IDO1 in both models but with stronger and more sustained expression in U251 cells, whereas U87 displayed a more gradual and delayed pattern. In parallel, distinct signaling adaptations emerged at the level of PI3K–AKT activity. In U87, chronic IFN-γ led to sustained AKT activation that continued after washout, suggesting engagement of PI3K–mTOR signaling to maintain metabolic resilience, consistent with oncogenic cross-talk described in GBM [28,29]. Interferon pathways have been reported to interface with PI3K/AKT signaling under prolonged cytokine tone, providing a plausible route for this adaptation [7,30].

Conversely, U251 cells exhibited durable AKT suppression with only partial recovery following cytokine washout and entered a sustained interferon-conditioned signaling and transcriptional state rather than a stable growth-suppressed state, including continued activation of IRDS-associated programs under chronic interferon exposure [31]. Consistent with this, population-level protein, transcriptomic, and secretome analyses indicate that IFN-γ washout does not uniformly restore signaling outputs to the untreated state. Instead, elements of interferon-conditioned pathway coordination persist after cytokine withdrawal, particularly in U87 cells, whereas U251 cells exhibit partial attenuation without full network reversion. Importantly, U251 cells resumed proliferation after IFN-γ washout despite incomplete restoration of pAKT and persistent suppression of pS6, indicating partial uncoupling between growth recovery and canonical PI3K–AKT–mTOR signaling outputs as assessed by these readouts. Notably, recovery of pAKT following cytokine washout occurred in the context of persistently reduced PI3K and pS6 levels, further supporting partial decoupling of canonical PI3K–AKT–mTOR signaling and suggesting the involvement of alternative regulatory inputs or incomplete restoration of mTORC1 activity. Although the PI3K–AKT–mTOR axis is often depicted as a linear cascade, phosphorylation at individual nodes can become uncoupled through feedback regulation and parallel inputs under chronic cytokine exposure.This polarity underscores that cytokine-receptor engagement of the PI3K–AKT–mTOR axis can generate opposing outputs [pro-survival or inhibitory] depending on network wiring and lineage context. The sustained expression of IRDS-associated genes in U251 echoes unphosphorylated-STAT-mediated persistence under chronic interferon tone, linking our observations to a clinically recognized, therapy-resistance signature [31]. Crosstalk between STAT and inflammatory circuits further stabilizes these states. STAT3 and NF-κB are frequently co-activated in tumors and have been reported to establish feed-forward inflammatory circuits that promote tumor progression while restraining antitumor immunity [32]. In GBM specifically, these axes have been implicated in mesenchymal/inflammatory programs and immune evasion [33,34], In this context, the coordinated phosphorylation patterns observed in our study may reflect engagement of similar signaling programs, although direct mechanistic interactions were not assessed. Consistent with this interpretation, U251 cells exhibited stronger STAT-associated inflammatory signatures and a chemokine-weighted, IRDS-like program, whereas U87 cells maintained a broader interferon-engaged yet immune-evasive equilibrium. Consistent with these signaling patterns, differences were also observed in the secretome. U87 cells displayed a broad pro-inflammatory output with TNF-α and CCL4 (MIP-1β), consistent with inflammatory signaling states that may be associated with non-clearing immune environments; CCR5-axis chemokines such as CCL4 are implicated in glioma progression and myeloid/T-cell trafficking [35]. In contrast, U251 skewed toward a CCL2-dominant profile with attenuation of multiple lymphokines (IL-2/4/5/10/13). CCL2 is a canonical recruiter of CCR2^+^ monocytes/M-MDSCs into GBM, reinforcing immunosuppressive myeloid circuits and therapy resistance [36]. The late rise in GM-CSF we observed is likewise consistent with reported roles in expansion and conditioning of suppressive myeloid populations in glioma [37].

Meanwhile, IL-10 down-modulation in U251 aligns with broader T-cell dysfunction paradigms in GBM, whereas persistent TNF-α/NF-κB signaling has been linked to stem-like programs and invasive behavior [38] The presence of IL-13 in U87 dovetails with the well-documented IL-13/IL-13Rα2 axis in GBM biology and therapeutics, underscoring lineage-specific cytokine wiring that may be actionable [39].

In this context, we emphasize that the molecular features highlighted below represent exploratory, context-dependent associations observed in vitro, rather than validated predictive or clinical biomarkers. Importantly, analysis of TCGA glioblastoma datasets supports the clinical relevance of these observations. Interferon-associated transcriptional programs were heterogeneous across tumors and were positively associated with PI3K–AKT pathway activity across molecular subtypes, consistent with the linkage between interferon signaling and survival pathways observed in vitro. In addition, elevated interferon signaling was associated with macrophage-enriched and T-cell exhaustion-associated signatures, reflecting an inflamed yet functionally suppressed immune microenvironment. Tumors with higher AKT pathway activity were also associated with reduced overall survival, suggesting a potential role of survival signaling in shaping interferon-associated tumor states. While these analyses are correlative and may be influenced by tumor purity as well as stromal or immune cell content that cannot be fully resolved in bulk transcriptomic datasets, they support the relevance of the adaptive programs identified in experimental models to human glioblastoma.

Taken together, these findings suggest that time-bounded interferon priming may capture transient antigen-presentation benefits while avoiding consolidation of PD-L1/IDO1 and IRDS programs [10,15,24,31]. The lineage-linked chemokine wiring observed here further supports biomarker-informed hypotheses for combination strategies, such as PD-1/PD-L1 or IDO1 inhibition in CCL4-dominant, checkpoint-inducible contexts, and CCR2/CCL2 axis modulation or JAK/STAT targeting in IRDS-persistent, CCL2-driven settings. In parallel, sustained PI3K–AKT engagement in the U87-like state suggests a potential vulnerability to co-targeting strategies aimed at disrupting survival-associated signaling, as PI3K/AKT co-inhibition may limit adaptive tumor persistence [40,41,42].

Our study is subject to several limitations. The in vitro, two-line model lacks myeloid and glial compartments and does not capture the full extent of patient-level heterogeneity characteristic of GBM. In addition, patient-derived glioblastoma models were not included. While analysis of TCGA datasets supports the relevance of the identified interferon-associated programs in human tumors, further validation in patient-derived and immune-competent models will be necessary to establish their generalizability and therapeutic implications. Furthermore, we did not perform targeted perturbation of key interferon-associated pathways, including STAT1/IRF1, IDO1, JAK, or AKT signaling. Nonetheless, the reproducible interferon imprinting, incomplete reversibility, and pathway bifurcations provide testable hypotheses for future co-culture and immune-competent experimental models. Because growth responses were assessed using longitudinal cell counts and doubling time calculations, additional orthogonal approaches (e.g., live-cell imaging or DNA synthesis assays) would further strengthen validation of proliferative trends. Finally, immune checkpoint gene expression and in vivo validation were not evaluated, which will be incorporated in future extensions of this work.

## 5. Conclusions

Chronic IFN-γ exposure does not impose a uniform tumoricidal program in glioblastoma but instead drives lineage-dependent adaptive states shaped by underlying signaling architecture. U87 cells acquire a persistence-prone, AKT-engaged state with delayed chemokine induction, whereas U251 cells adopt an IRDS-like, AKT-suppressed, CCL2-dominant program. These adaptive responses are only partially reversible following cytokine washout, consistent with sustained interferon imprinting. Integration with human glioblastoma datasets further supports the clinical relevance of these states, linking interferon-associated transcriptional programs to survival signaling and immune context. Together, these findings highlight how chronic immune signaling can reprogram tumor cell behavior in a context-dependent manner and underscore the importance of considering temporal and lineage-specific responses when designing interferon-based or combination therapeutic strategies in glioblastoma.

## Data Availability

The datasets supporting the conclusions of this article are available in the Gene Expression Omnibus (GEO) repository, https://www.ncbi.nlm.nih.gov/geo/query/acc.cgi?acc=GSE304761, accessed on 12 August 2025. The code used for data analysis and figure generation is available upon request from the corresponding authors.

## Supplementary Materials

The following supporting information can be downloaded at: https://www.mdpi.com/article/10.3390/cancers18101552/s1, Figure S1: Quality control and overview of RNA-seq analysis in U87 glioblastoma cells following IFN-γ treatment; Figure S2. Quality control and overview of RNA-seq analysis in U251 glioblastoma cells following IFN-γ treatment; Figure S3: Checkpoint-associated transcriptional responses to chronic IFN-γ exposure in glioblastoma cell lines. Table S1. Cell Growth and Proliferation Statistics for U87 and U251 Cells under IFN-γ Treatment. Table S2. Automated Western Pseudo Blots Quantification and Phosphoprotein Signaling Analysis across IFN-γ Treatment Time Course; Table S3. RNA-Seq Analysis Summary for U87 Cells under IFN-γ Treatment; Table S4. RNA-Seq Analysis Summary for U251 Cells under IFN-γ Treatment; Table S5. Integrated Summary of Functional, Transcriptomic, and Signaling Responses to Chronic IFN-γ Exposure in U87 and U251 Cells.

## Abbreviations

The following abbreviations are used in this manuscript:

AKT	Protein kinase B
ANOVA	Analysis of variance
B2M	Beta-2-microglobulin
BCA	Bicinchoninic acid
BSA	Bovine serum albumin
CCL	C-C motif chemokine ligand
CCR2	C-C chemokine receptor type 2
CIITA	Class II transactivator
CNS	Central nervous system
CXCL	C-X-C motif chemokine ligand
DEG	Differentially expressed gene
DMEM	Dulbecco’s Modified Eagle Medium
DMSO	Dimethyl sulfoxide
DTT	Dithiothreitol
ELISA	Enzyme-linked immunosorbent assay
ERK	Extracellular signal-regulated kinase
FBS	Fetal bovine serum
FDR	False discovery rate
FGF	Fibroblast growth factor
GBM	Glioblastoma multiforme
GEO	Gene Expression Omnibus
GO	Gene Ontology
GRCh38	Genome Reference Consortium Human Build 38
IFN-γ	Interferon gamma
IFNGR	Interferon gamma receptor
IL	Interleukin
IRDS	Interferon-related DNA-damage resistance signature
IRF	Interferon regulatory factor
ISG	Interferon-stimulated gene
JAK	Janus kinase
MAPK	Mitogen-activated protein kinase
MHC	Major histocompatibility complex
mTOR	Mechanistic target of rapamycin
NF-κB	Nuclear factor kappa-light-chain-enhancer of activated B cells
NK	Natural killer (cell)
PANTHER	Protein Analysis Through Evolutionary Relationships
PBS	Phosphate-buffered saline
PD-1	Programmed cell death protein 1
PD-L1	Programmed death ligand 1
PI3K	Phosphoinositide 3-kinase
PSMB	Proteasome subunit beta
PSME	Proteasome activator subunit
qPCR	Quantitative polymerase chain reaction
RIN	RNA integrity number
RRID	Research Resource Identifier
RNA-seq	RNA sequencing
RIPA	Radioimmunoprecipitation assay buffer
SD	Standard deviation
S6	Ribosomal protein S6
SOCS	Suppressor of cytokine signaling
STAT	Signal transducer and activator of transcription
TCGA	The Cancer Genome Atlas
TNF-α	Tumor necrosis factor alpha
WES	Wes automated capillary Western system (ProteinSimple)
WO	Washout
