# Supplementary material for: Chronic IFN-γ Exposure Induces Divergent Adaptive Programs in Glioblastoma Subtypes
Source: Cancers (Basel). 2026 May 11;18(10):1552. doi: 10.3390/cancers18101552 (PMC13204958; doi:10.3390/cancers18101552)
Supplement: Supplementary file 1 [file cancers-18-01552-s001.zip › cancers-4260457-Supplementary Material/Data Summary Table-S5.pdf]

**Functional category**	**Readout type**	**Marker / pathway**	**Time context**	**U87 response**	**U251 response**
IFN signaling	Protein	pSTAT1	Early → chronic	Activated	Activated
IFN transcriptional program	RNA-seq	Interferon response genes	Across time course	Biphasic (on–off–on)	Sustained (IRDS-like)
PI3K–AKT signaling	Protein	pAKT	Chronic / post-washout	Sustained activation (persists after washout)	Suppressed
mTOR signaling	Protein	pS6	Chronic	Sustained activation	Suppressed
Immune checkpoints	RNA-seq	CD274 (PD-L1)	Chronic	Progressive induction	Strong, sustained induction
Immune checkpoints	RNA-seq	IDO1	Chronic (delayed onset)	Delayed induction	Strong, sustained induction
Secretome profile	Cytokine panel	Global secretion pattern	Chronic	Broad poly-cytokine profile	Selective TNFα/CCL2-dominant profile
Signaling network organization	Single-Cell Proteomics	Pathway coordination	Chronic	Diffuse network-wide amplification	Focused signaling axis (STAT3–NF-κB)
Growth phenotype	Functional	Proliferation during IFN-γ	Chronic	Reduced	Reduced
Post-treatment recovery	Functional	Proliferation after washout	Washout	Partial recovery	Full recovery
IFN memory state	Transcriptomic	Post-washout persistence	Washout	Partial reversal	Persistent/intermediate retention
