# Supplementary material for: Chronic IFN-γ Exposure Induces Divergent Adaptive Programs in Glioblastoma Subtypes
Source: Cancers (Basel). 2026 May 11;18(10):1552. doi: 10.3390/cancers18101552 (PMC13204958; doi:10.3390/cancers18101552)
Supplement: Supplementary file 1 [file cancers-18-01552-s001.zip › cancers-4260457-Supplementary Material/Supplementary Figures.pdf]

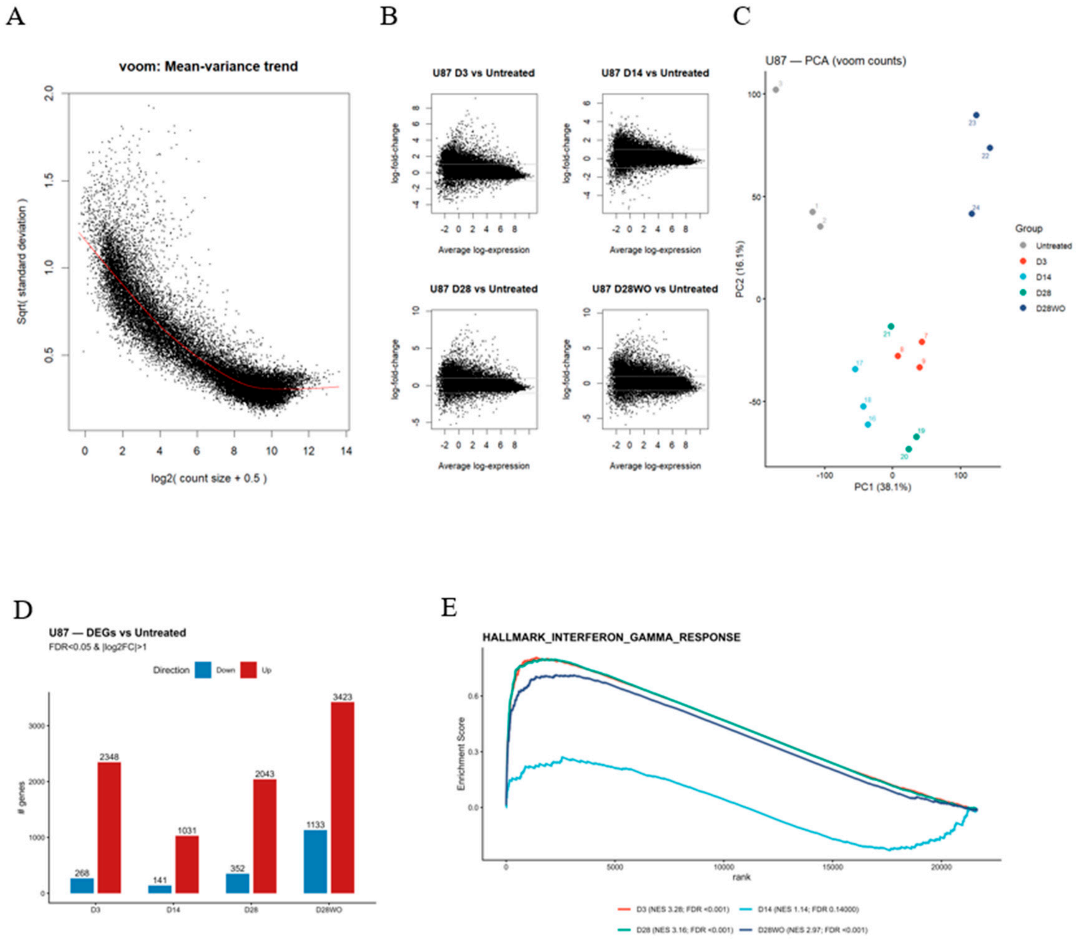

Figure S1. Quality control and overview of RNA-seq analysis in U87 glioblastoma cells following IFN-γ treatment. (**A**) Mean–variance trend (voom): The fitted mean–variance relationship from limma-voom demonstrates appropriate modeling of heteroscedasticity across the dynamic range of log_2-transformed counts. (**B**) MA plots: Log_2 fold change versus average log-expression for each comparison (D3, D14, D28, D28WO vs Untreated) illustrating genome-wide distribution of differentially expressed genes (DEGs) at each time point. (**C**) Principal component analysis (PCA): Separation of transcriptomes by treatment duration, showing distinct clustering of early versus late time points. Replicates at D28 and D28WO exhibit broader dispersion, indicating increased transcriptional heterogeneity after prolonged IFN-γ exposure. (**D**) Number of DEGs: Bar plot showing up- and down-regulated genes (FDR < 0.05, $|log_2FC| > 1$) at each time point compared to Untreated. The number of DEGs increases progressively with treatment duration and partially reverses after IFN-γ withdrawal. (**E**) GSEA enrichment plots: Enrichment of the Hallmark Interferon Gamma Response gene set confirming persistent activation of IFN-γ

signaling across all time points, with the strongest enrichment observed at D3 and D28.

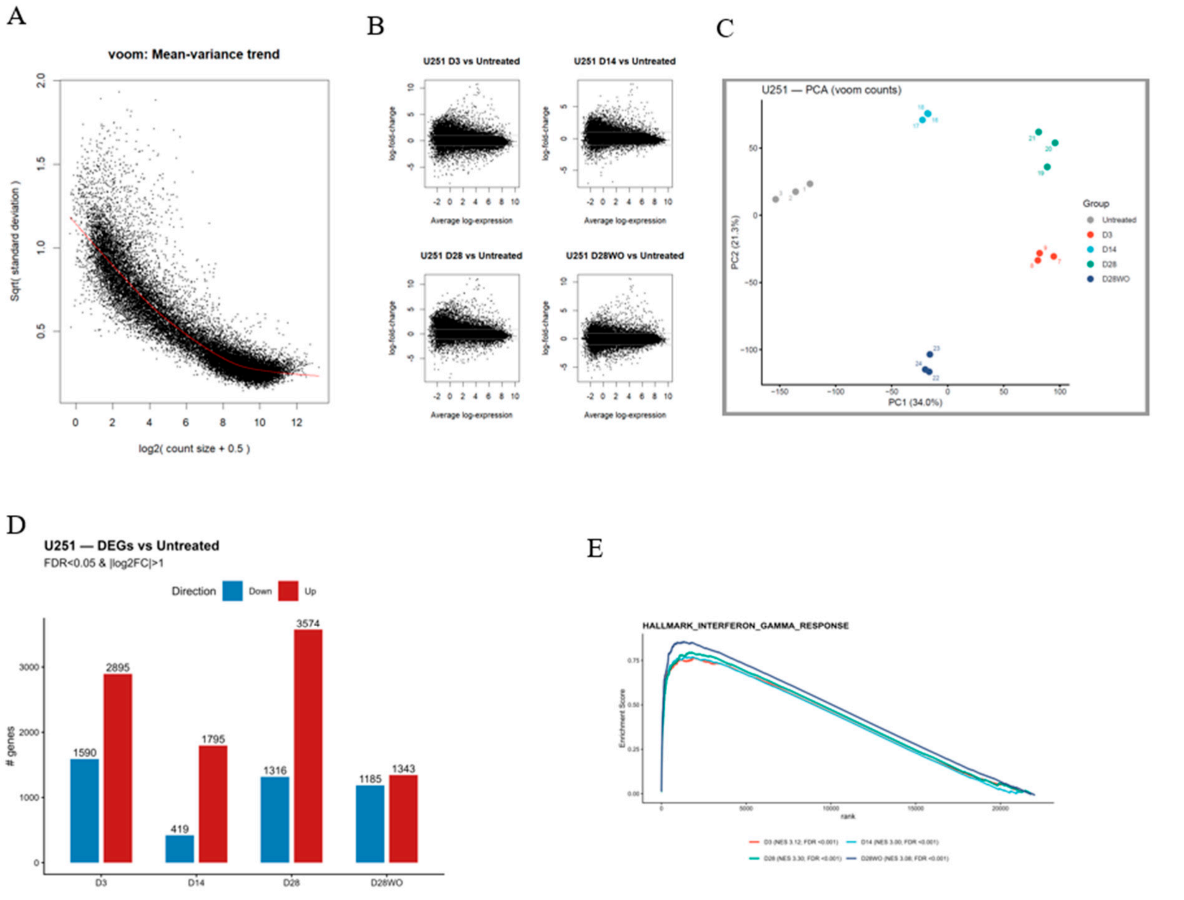

Figure S2. Quality control and overview of RNA-seq analysis in U251 glioblastoma cells following IFN-γ treatment. (**A**) Mean–variance trend (voom): Fitted mean–variance relationship showing proper modeling of heteroscedasticity across $\log_2$-transformed counts before differential analysis using limma-voom. (**B**) MA plots: Log_2 fold-change versus average log-expression for each comparison (D3, D14, D28, D28WO vs Untreated), displaying genome-wide gene expression changes at each time point. (**C**) Principal component analysis (PCA): Clustering of samples by treatment group, demonstrating distinct transcriptomic separation following IFN-γ exposure. U251 replicates show tighter clustering compared to U87, indicating a more uniform response across time points. (**D**) Number of DEGs: Bar plot summarizing up- and down-regulated genes ($FDR < 0.05$, $|\log_2 FC| > 1$) relative to Untreated controls. The total number of regulated genes increases over time and remains elevated after washout, reflecting the persistence of IFN-γ–driven transcriptional changes. (**E**) GSEA enrichment plots: Enrichment of the Hallmark Interferon Gamma Response gene set across all time points, confirming sustained activation of IFN-γ–responsive programs throughout treatment and partial maintenance after withdrawal.

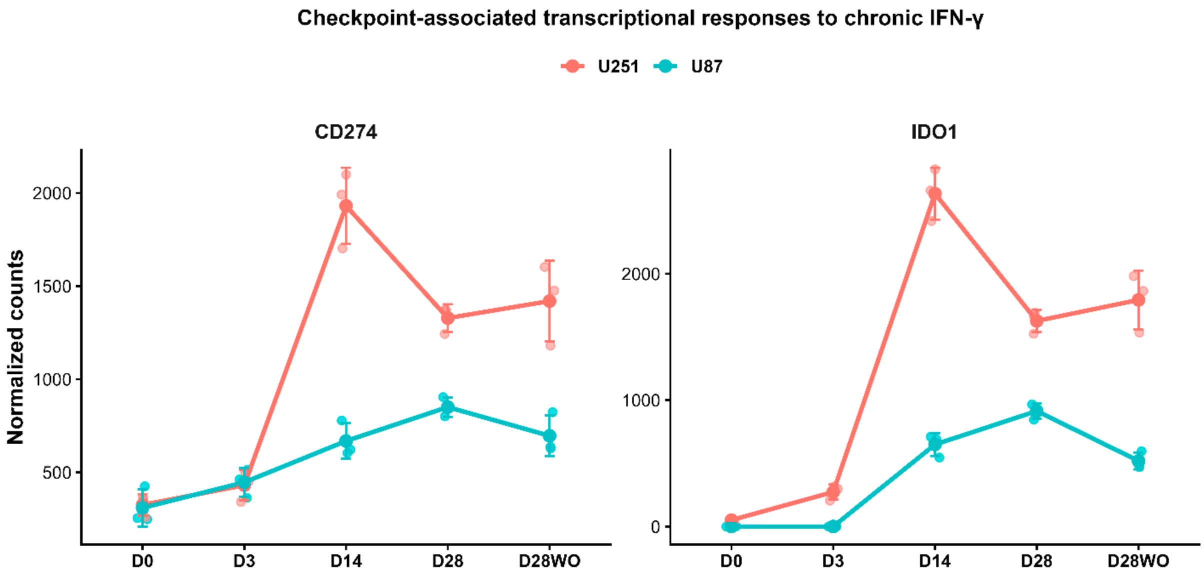

Figure S3. Checkpoint-associated transcriptional responses to chronic IFN-γ exposure in glioblastoma cell lines. Normalized RNA-seq counts for CD274 (PD-L1) (left) and IDO1 (right) in U87 and U251 cells across the treatment time course (D0, D3, D14, D28, and D28WO). Cells were exposed to IFN-γ (10 ng/mL) for up to 28 days, followed by cytokine washout (WO) where indicated. Data are shown as mean ± SEM from n = 3 independent biological replicates, with individual replicates overlaid. Both genes are induced in response to IFN-γ in both models, with distinct temporal dynamics. U251 cells exhibit higher baseline expression and stronger, sustained induction, whereas U87 cells display more gradual and moderate increases. These findings are consistent with lineage-dependent differences in interferon-driven immune checkpoint regulation.
